# Risk Stratification and Treatment Selection in Patients With Asymptomatic Abdominal Aortic Aneurysms

**DOI:** 10.1001/jamanetworkopen.2025.3559

**Published:** 2025-04-07

**Authors:** Lorenz Meuli, Alexander Zimmermann, Jeppe Kofoed Petersen, Emil Loldrup Fosbøl, Vaiva Dabravolskaité, Vladimir Makaloski, Jonas Peter Eiberg, Lars Valeur Køber, Timothy Andrew Resch

**Affiliations:** 1 Department of Vascular Surgery, University Hospital Zurich, University of Zurich, Zurich, Switzerland; 2 Department of Vascular Surgery, The Heart Center, University Hospital Copenhagen Rigshospitalet, Copenhagen, Denmark; 3 Department of Clinical Medicine, Faculty of Health and Medical Sciences, University of Copenhagen, Copenhagen, Denmark; 4 Department of Cardiology, The Heart Center, University Hospital Copenhagen Rigshospitalet, Copenhagen, Denmark; 5 Department of Vascular Surgery, University of Bern, Inselspital, Bern, Switzerland; 6 Copenhagen Academy for Medical Education and Simulation (CAMES), Copenhagen, Denmark

## Abstract

**Importance:**

Open surgical repair (OSR) should be prioritized for patients with asymptomatic abdominal aortic aneurysm (AAA) and long life expectancy, whereas endovascular repair (EVAR) is preferred for patients with suitable anatomy and life expectancy less than 2 to 3 years. However, life expectancy estimation and risk stratification are not well established.

**Objective:**

To evaluate risk-stratified survival differences between OSR and EVAR following elective AAA treatment.

**Design, Setting, and Participants:**

This cohort study used data from Danish national health registries. Patients older than 60 years undergoing elective AAA repair between 2004 and 2023 were categorized into 4 risk groups according to age, estimated glomerular filtration rate, and chronic obstructive pulmonary disease. Follow-up was until March 31, 2024.

**Exposure:**

OSR or EVAR for AAA.

**Main Outcomes and Measures:**

The primary outcome was overall survival. Secondary outcomes were incidence of AAA rupture and new cancer diagnosis. Comorbidities were balanced using inverse probability weighting. Kaplan-Meier estimators were generated for both treatments and the 4 risk score groups.

**Results:**

Of 6891 identified patients, 5757 (83.4%) were men. Women were older (median [IQR] age, 75.4 [70.9-79.3] vs 74.5 [70.5-78.5] years), more often had chronic obstructive pulmonary disease (156 women [13.6%] vs 512 men [8.9%]), and had lower estimated glomerular filtration rate (median [IQR], 68.4 [54.2-80.4] vs 70.4 [56.5-82.4] mL/min/1.73 m^2^) compared with men. The median follow-up was 8.28 years (95% CI, 8.10-8.50 years). OSR was associated with higher perioperative mortality in all risk groups. In low-risk patients, OSR was associated with a 10-month (95% CI, 2.2-18.3 months; *P* = .02) longer mean survival time restricted at 15 years compared with EVAR. In moderate-to-high-risk patients, OSR was associated with a 9-month (95% CI, 1.9-16.9 months; *P* = .008) shorter mean survival time restricted after 12.5 years compared with EVAR. No difference in mean survival time was seen in low-to-moderate-risk and high-risk patients at the study end. No differences in 10-year incidence of secondary AAA ruptures (OSR, 2.6% [95% CI, 1.9%-3.4%] vs EVAR, 2.2% [95% CI, 1.7%-2.7%]; *P* = .34) or solid malignant tumor (OSR, 18.6% [95% CI, 16.7%-20.5%] vs EVAR, 20.5% [95% CI, 18.9%-22.1%]; *P* = .35) were detected.

**Conclusions and Relevance:**

In this cohort study of 6891 patients with AAA, OSR was associated with higher perioperative mortality in all risk groups, but with longer mean survival only in low-risk patients. Conversely, EVAR was associated with longer mean survival in moderate-to-high-risk patients. These findings highlight the potential benefits of risk stratification when planning AAA treatment.

## Key Points

**Question:**

Which treatment for abdominal aortic aneurysms, open repair or endovascular repair, is associated with better long-term survival?

**Findings:**

In this cohort study of 6891 patients with abdominal aortic aneurysms stratified into 4 risk groups, open repair was associated with significantly better long-term survival in low-risk patients, whereas endovascular repair was associated with significantly better long-term survival in moderate-to-high-risk patients. No statistically significant survival differences were seen in the other risk groups.

**Meaning:**

This study highlights the potential benefits of risk stratification when planning abdominal aortic aneurysm treatment.

## Teaser

This cohort study evaluates risk-stratified survival differences between open surgical repair and endovascular repair following elective treatment for abdominal aortic aneurysm.

## Introduction

The choice of surgical technique for elective abdominal aortic aneurysms (AAAs) is a challenge because multiple factors should be considered when individualizing treatment. The 2018 Society for Vascular Surgery practice guidelines on the care of patients with an AAA recommend using a detailed score to estimate the perioperative mortality but do not further detail estimation of life expectancy or choice of treatment based on risk profile.^1^ The 2024 clinical practice guidelines of the European Society for Vascular Surgery recommend that physiological reserves and life expectancy be the primary determinants when choosing repair option.^2^ Open surgical repair (OSR) should be considered as the preferred treatment modality for most patients with long life expectancy, defined as greater than 10 to 15 years.^2^ In patients with a limited life expectancy of less than 2 to 3 years, elective AAA repair is not recommended.^2^ For patients with a life expectancy of 3 to 15 years, the European guidelines recommend that endovascular aneurysm repair (EVAR) should be considered as the preferred treatment modality if anatomically suitable.^2^ These recommendations reflect the fact that the perioperative mortality is significantly lower with EVAR compared with OSR,^3,4^ but the benefits for EVAR diminish over time, leading to long-term equipoise in overall survival,^5^ or even long-term superiority for OSR.^6^ There is consensus that risk stratification and estimation of life expectancy in patients with asymptomatic AAA is central when considering elective repair.^1,2^ However, robust data to support risk-stratified clinical decision-making for elective AAA repair are largely missing.

Our study group previously developed and validated a risk score to estimate survival following elective EVAR in patients with asymptomatic AAA.^7,8^ Age, estimated glomerular filtration rate (eGFR), and chronic obstructive pulmonary disease (COPD) were selected using least absolute shrinkage and selection operator, and a risk score was calculated using a cohort of 558 patients at the University Hospital in Bern, Switzerland.^7^ Discrimination ability of the risk score was good (Harrel C, 0.70; 95% CI, 0.66-0.75), and internal validation confirmed robustness. The resulting risk score with 4 risk groups was then externally validated on 1500 patients from 4 European centers (Turku and Helsinki, Finland; Zurich, Switzerland; and Leuven, Belgium). External validation of the risk score in an international cohort of demonstrated slightly reduced discrimination ability (Harrel C, 0.62; 95% CI, 0.60-0.65) between groups. The 5-year survival rates were 40% for high-risk patients, 68% for moderate-to-high-risk patients, 83% for low-to-moderate-risk patients, and 89% for low-risk patients. Calibration revealed that the estimated vs observed overall survival rates were 69.5% vs 70.3% at 5 years and 37.0% vs 38.3% at 10 years.^8^

The aim of this study was to assess differences in long-term survival between OSR and EVAR for nonruptured AAA across different risk groups. In addition, we sought to identify new oncological and cardiovascular diagnoses during follow-up.

## Methods

This population-based cohort study used Danish registries that are linkable with the Danish Civil Registration Number given to all Danish citizens at birth.^7^ In Denmark, registry-based studies that are conducted for the sole purpose of statistics and scientific research do not require ethical approval or informed consent by law. However, this study was approved by the Capital Region of Denmark in accordance with the General Data Protection Regulation and is reported according to the Strengthening the Reporting of Observational Studies in Epidemiology (STROBE) reporting guidelines.^9,10^ In accordance with the rules on anonymity of Statistics Denmark, all personal identifiers were anonymized, and subclassifications with 5 or fewer patients were not reported.

### Data Source

The Danish National Patient Registry contains data on all admissions to Danish hospitals and captures 1 mandatory primary diagnosis and multiple secondary diagnoses for each admission, as well as performed medical procedures and death records. Diagnoses are coded according to the *International Statistical Classification of Diseases and Related Health Problems, Tenth Revision (ICD-10)*, and medical procedures are coded according to the Danish version of the Nordic Classification of Surgical Procedures. Comorbidities and secondary outcomes were defined on the basis of the validated items of the Elixhauser score.^11^ The Danish National Patient Registry was used to identify all patients older than 60 years treated for nonruptured AAA (*ICD-10* code I71.4) between January 1, 2004, and December 31, 2023, to extract comorbidities at the time of treatment, and to extract diagnoses given during follow-up, as well as date of death. Only patients with a surgical code of an infrarenal AAA repair were included. The Danish Population Registry was used to link date of birth and sex. The Register of Pharmaceutical Sales was used to link data on all filled prescriptions for COPD prior to surgery using the Anatomical Therapeutic Chemical classification. The Clinical Laboratory Information Register was used to link serum creatinine levels prior to surgery. No restrictions were placed on the time interval between sampling and the procedure. The codes used in this study are detailed in eTable 1 in Supplement 1. Information was extracted from the registries on March 31, 2024. The data sources have been extensively used for research purposes and have been described in detail previously.^9,12,13,14^

### Outcomes

The primary outcome was overall survival. Secondary outcomes included incidence rates of cardiovascular diagnosis (ie, new diagnosis of atrial fibrillation, coronary artery disease, myocardial infarction, heart failure, stroke, secondary AAA rupture, dialysis, COPD, or diabetes), as well as oncological diagnosis (ie, new diagnosis of lymphoma, solid tumor, or metastasis).

### Risk Stratification

**Figure 1.**
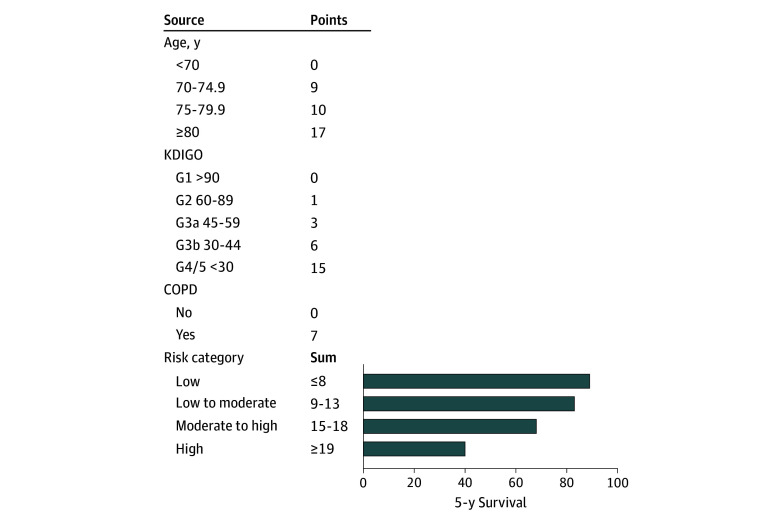
Risk Score Risk score to estimate 5-year survival for patients with asymptomatic abdominal aortic aneurysms according to Meuli et al^7^ and external validation by Dabravolskaité et al.^8^ Five-year survival was 89% in the low-risk group, 83% in the low-to-moderate-risk group, 68% in the moderate-to-high-risk group, and 40% in the high-risk group. Numbers with Kidney Disease: Improving Global Outcomes (KDIGO) categories refer to estimated glomerular filtration rates in milliliters per minute per 1.73 meters squared. COPD indicates chronic obstructive pulmonary disease.

A validated risk score was used to stratify the cohort into 4 risk groups.^7,8^ The risk score is based on the variables age, eGFR, and COPD at the time of treatment and is detailed in Figure 1. The score identified low-risk patients (≤8 points), low-to-moderate-risk patients (9-13 points), moderate-to-high-risk patients (15-18 points), and high-risk patients (≥19 points).

### Statistical Analysis

Imbalances between characteristics of patients within the 4 risk group were addressed using propensity score weighting. Least absolute shrinkage and selection operator was used to select baseline variables for propensity score estimation, with the optimal penalty parameter determined via cross-validation.^15^ The selected variables were age, sex, COPD, atrial fibrillation, heart failure, myocardial infarction, diabetes, solid malignant tumor, and date of treatment. A logistic regression model with the type of treatment as dependent variable and the selected independent variables was built to estimate propensity scores and assign inverse probability weights (IPWs).^16,17^ Balance was assessed using standardized mean differences, with values less than 0.2 indicating good balance.^18^ Details on the variable selection process and the IPW balance are illustrated in eFigures 1, 2, and 3 in Supplement 1.

IPW Kaplan-Meier estimators were generated for both treatments and the 4 risk score groups. As nonproportionality was identified, survival times were analyzed using restricted mean survival time (RMST).^19,20^ The 95% CIs were estimated using the percentile method on 10 000 bootstrap resamples. Differences in RMST between OSR and EVAR was plotted over time using penalized regression smoothers.

Unadjusted and IPW cumulative incidence rates for diagnosis during follow-up were estimated for both types of treatment, with death as a competing risk, and were compared using the Gray test.^21^ The statistical analysis was performed using R Studio version 4.2.1 (R Project for Statistical Computing) on a Windows Server 2021 on a secured server environment in Denmark. Statistical significance was defined as 2-sided *P* < .05.

## Results

Between 2004 and 2023, 10 852 patients older than 60 years underwent infrarenal repair of a nonruptured AAA in Denmark. After excluding 3961 patients without preoperative creatinine measurements available, 6891 (5757 men [83.4%]) remained for analysis, with 3926 (57.0%) undergoing OSR and 2965 (43.0%) treated with EVAR. The proportion of patients undergoing OSR decreased from 86.5% (409 of 473 patients) in 2004 to 2008 to 47.2% (1147 of 2429 patients) in 2019 to 2023. Missing creatinine data were more common in the early years, predominantly affecting patients undergoing OSR (2828 patients [41.9%]) compared with those undergoing EVAR (525 patients [15.0%]).

Table 1 shows baseline characteristics of the 6891 analyzed patients by risk group. At the time of treatment, women were significantly older (median [IQR] age, 75.4 [70.9-79.3] vs 74.5 [70.5-78.5] years), more often had COPD (156 women [13.6%] vs 512 men [8.9%]), and had lower eGFR (median [IQR], 68.4 [54.2-80.4] vs 70.4 [56.5-82.4] mL/min/1.73 m^2^) compared with men. Unadjusted and weighted baseline characteristics for each risk group, stratified by treatment, are provided in eTables 2, 3, 4, and 5 in Supplement 1.

**Table 1.** Baseline Characteristics by Risk Score Group^a^

Characteristic	Patients, No. (%)				
	Low risk (n = 1438)	Low-to-medium risk (n = 3366)	Medium-to-high risk (n = 1216)	High risk (n = 871)	Overall (N = 6891)
Age, median (IQR), y	67 (65-69)	75 (72-77)	80 (75-83)	82 (80-84)	75 (71-79)
Sex					
Male	1222 (85.0)	2846 (84.6)	990 (81.4)	689 (79.1)	5747 (83.4)
Female	216 (15.0)	520 (15.4)	226 (18.6)	182 (20.1)	1144 (16.6)
Estimated glomerular filtration rate, median (IQR), mL/min/1.73 m^2^	74 (63-84)	74 (62-85)	70 (45-80)	48 (38-55)	70 (56-82)
Chronic obstructive pulmonary disease	114 (7.9)	32 (1.0)	266 (21.9)	256 (29.4)	668 (9.7)
Atrial fibrillation	96 (6.7)	368 (10.9)	155 (12.7)	142 (16.3)	761 (11.0)
Coronary artery disease	307 (21.3)	692 (20.6)	281 (23.1)	212 (24.3)	1492 (21.7)
Myocardial infarction	206 (14.3)	432 (12.8)	162 (13.3)	125 (14.4)	925 (13.4)
Heart failure	93 (6.5)	205 (6.1)	104 (8.6)	120 (13.8)	522 (7.6)
Diabetes	130 (9.0)	308 (9.2)	131 (10.8)	115 (13.2)	684 (9.9)
Stroke	174 (12.1)	494 (14.7)	180 (14.8)	150 (17.2)	998 (14.5)
Solid tumor	174 (12.1)	540 (16.0)	208 (17.1)	159 (18.3)	1081 (15.7)
Lymphoma	18 (1.3)	39 (1.2)	19 (1.6)	15 (1.7)	91 (1.3)
Period of treatment					
2004-2008	145 (10.1)	186 (5.5)	77 (6.3)	65 (7.5)	473 (6.9)
2009-2013	369 (25.7)	761 (22.6)	224 (18.4)	179 (20.6)	1533 (22.2)
2014-2018	511 (35.5)	1246 (37.0)	417 (34.3)	282 (32.4)	2456 (35.6)
2019-2023	413 (28.7)	1173 (34.8)	498 (41.0)	345 (39.6)	2429 (35.2)

^a^ The risk score to estimate long-term survival for patients with asymptomatic abdominal aortic aneurysm according to Figure 1 was used for grouping.^7,8^

### Perioperative Mortality

The raw and adjusted 30-day mortality rates by risk group and mode of treatment are presented in Table 2. In low-risk patients, the adjusted 30-day mortality rate was 1.8% (95% CI, 1.0%-2.6%) for OSR compared with 1.1% (95% CI, 0.3%-1.9%) for EVAR. In high-risk patients, the adjusted 30-day mortality rate was 16.2% (95% CI, 12.3%-20.0%) for OSR compared with 2.1% (95% CI, 0.8%-3.4%) for EVAR.

**Table 2.** Thirty-Day Mortality for Elective Abdominal Aortic Aneurysm Repair by Treatment and Risk Group

Repair and risk group	No. of deaths	No. of patients at risk	Mortality, % (95% CI)	
			Raw	Weighted
Open repair				
Low	19	986	1.9 (1.0-2.7)	1.8 (1.0-2.6)
Low to medium	96	1856	4.9 (3.9-5.9)	5.1 (4.0-6.1)
Medium to high	53	526	9.1 (6.8-11.4)	9.5 (6.9-12.1)
High	65	312	17.2 (13.3-20.9)	16.2 (12.3-20.0)
Overall	233	3680	5.9 (5.2-6.7)	6.6 (5.7-7.4)
Endovascular aneurysm repair				
Low	7	421	1.6 (0.4-2.8)	1.1 (0.3-1.9)
Low to medium	11	1386	0.8 (0.3-1.2)	0.7 (0.3-1.1)
Medium to high	10	619	1.6 (0.6-2.5)	1.5 (0.5-2.4)
High	10	481	2.0 (0.8-3.3)	2.1 (0.8-3.4)
Overall	38	2907	1.3 (0.9-1.7)	1.1 (0.7-1.4)

### Long-Term Survival

**Figure 2.**
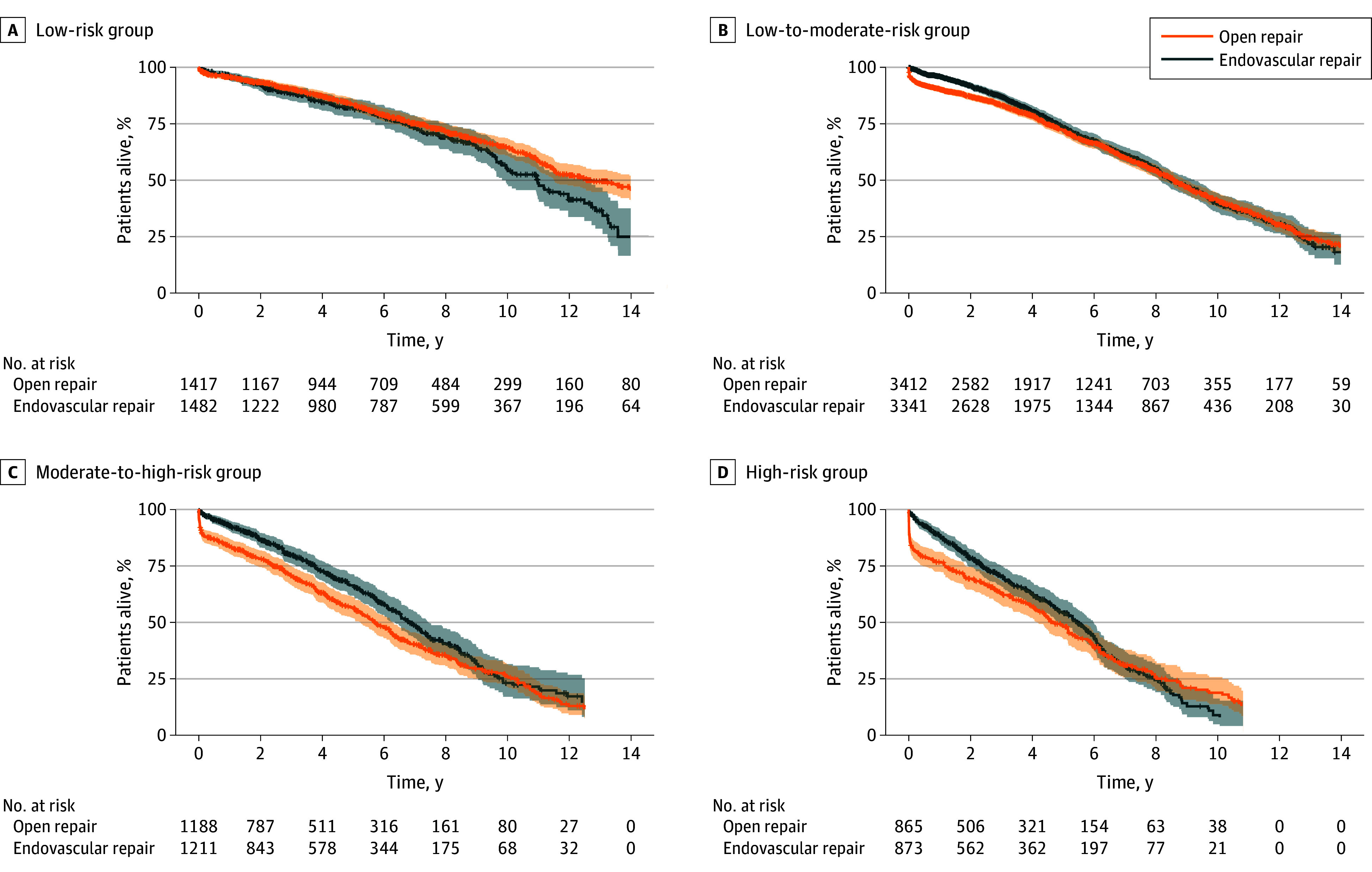
Survival Curves for Elective Abdominal Aortic Aneurysm Repair by Treatment and Risk Group Adjusted Kaplan-Meier curves show overall survival after elective treatment of intact abdominal aortic aneurysms stratified by risk score and type of treatment. Numbers at risk reflect the weighted pseudopopulation. Shaded areas denote 95% CIs. Estimators were trimmed at 15 years and if the number at risk was less than 20 in any group. The risk score is presented in Figure 1.

The median follow-up time was 8.28 years (95% CI, 8.10-8.50 years), with a total of 2983 deaths recorded. The adjusted 10-year survival rate was 41.6% (95% CI, 39.6%-43.8%) after OSR and 38.0% (95% CI, 35.0%-41.2%) after EVAR (*P* = .19). Raw and adjusted survival rates at 1, 5, and 10 years are provided in eTable 6 in Supplement 1. Figure 2 shows adjusted treatment-specific KM survival curves stratified by the 4 risk groups. The observed differences in perioperative mortality are reflected in early differences in the KM estimators (Figures 2B, 2C, and 2D). In the long-term follow-up, the observed associations for higher early survival after EVAR were gradually lost with crossing survival curves after 6 to 10 years. Consequently, the hazard rates for death during the follow-up were nonproportional between the 2 treatments.

**Figure 3.**
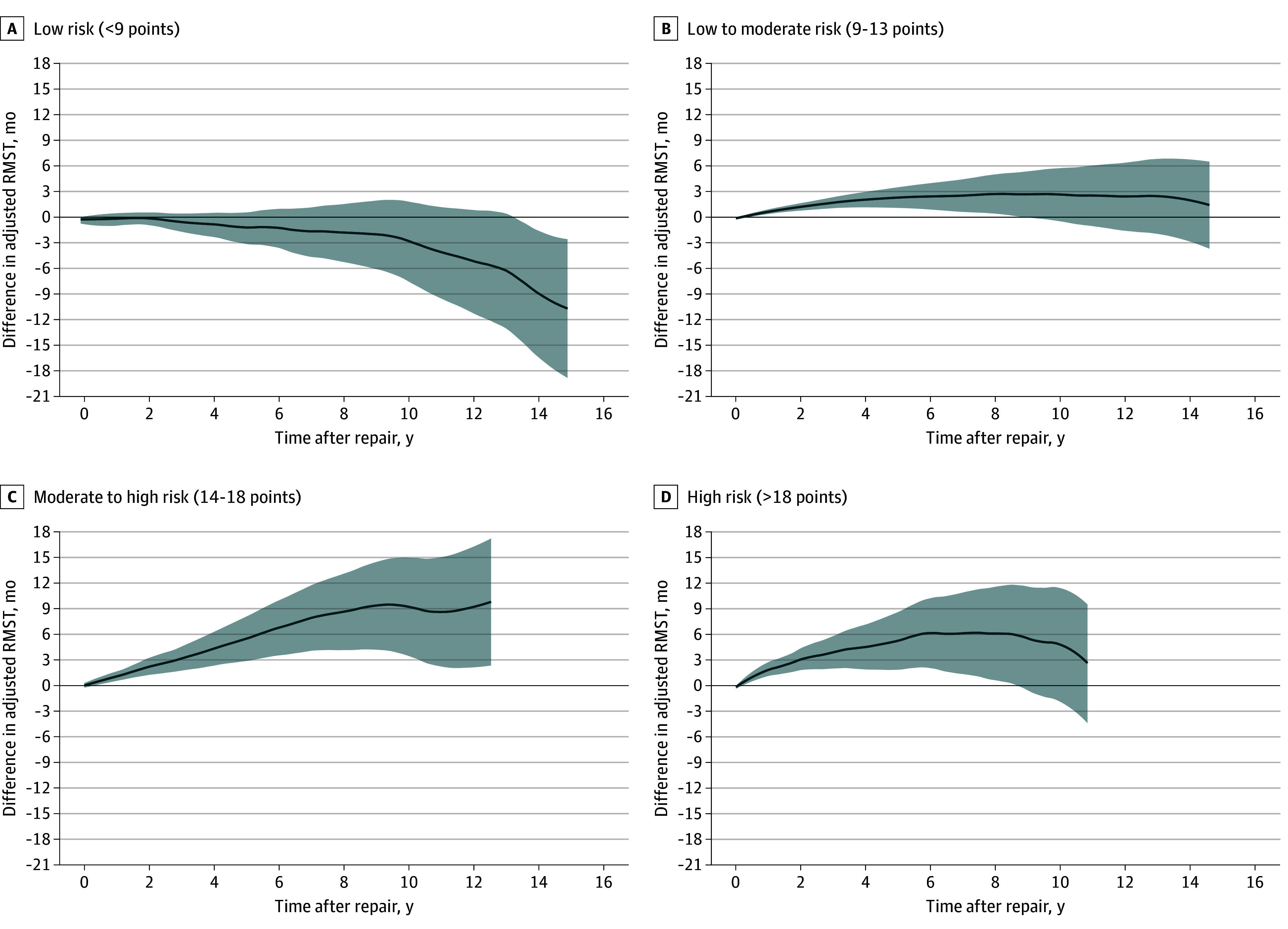
Differences in Survival Time for Elective Abdominal Aortic Aneurysm Repair by Treatment and Risk Group Differences in restricted mean survival time (RMST) after elective treatment of abdominal aortic aneurysms. Graphs show the risk groups as specified in Figure 1 and detailed in eTables 2 to 5 in Supplement 1. Estimators were trimmed at 15 years and if the number at risk was less than 20 (Figure 2). Shaded areas denote 95% CIs. For example, the mean survival time in low-risk patients restricted at 15 years of follow-up was 10 months (95% CI, 2.2-18.3 months) longer if treated with open repair compared with endovascular repair.

Figure 3 illustrates risk-stratified differences in RMST during follow-up between treatments. In low-risk patients (Figure 3A), EVAR was associated with a significantly shorter RMST of 10.0 months at 15 years (95% CI, 2.2-18.3 months; *P* = .02) compared with OSR. In low-to-moderate-risk patients (Figure 2B), EVAR had a significantly higher early RMST, which diminished over time, becoming nonsignificant after 8.9 years. In moderate-to-high-risk patients (Figure 2C), EVAR’s early advantage in RMST persisted, resulting in a 9-month longer RMST at 12.5 years (95% CI, 1.9-16.9 months; *P* = .008). For high-risk patients (Figure 2D), EVAR’s early RMST advantage became nonsignificant after 8.7 years.

### Diagnosis During Follow-Up

Cumulative incidence rates of secondary AAA ruptures at 10 years were 2.6% (95% CI, 1.9%-3.4%) after OSR and 2.2% (95% CI, 1.7%-2.7%) after EVAR (*P* = .34). A significantly higher incidence of strokes was recorded in patients after EVAR with 14.0% (95% CI, 12.7%-15.3%) compared with 10.9% (95% CI, 9.4%-12.4%) after OSR (*P* < .001). On the other hand, new diagnosis of heart failure was recorded more often after OSR in 15.0% (95% CI, 13.3%-16.8%) of patients after 10 years compared with 13.0% (95% CI, 11.7%-14.4%) of patients undergoing EVAR (*P* = .005). There was no statistically significant difference for cumulative incidence rates of solid malignant tumors (OSR vs EVAR, 18.6% [95% CI, 16.7%-20.5%] vs 20.5% [95% CI, 18.9%-22.1%]; *P* = .35), lymphoma, or tumor metastasis, as well as new-onset of atrial fibrillation, coronary artery disease, myocardial infarction, dialysis, diabetes, or COPD between patients treated with OSR and EVAR. eFigure 4 in Supplement 1 shows the adjusted cumulative incidence of first-time cardiovascular and oncological diagnosis during follow-up after AAA treatment. Of note, approximately 15.7% of patients already had a tumor diagnosis at the time of AAA treatment.

## Discussion

This cohort study contributes to a better understanding of perioperative mortality and long-term survival of patients treated for nonruptured AAA. Table 2 demonstrates that the perioperative mortality for OSR was associated with patients’ risk profile with a prohibitively high mortality rate in the high-risk cohort of 16.2% (95% CI, 12.3%-20.0%). In contrast, OSR had comparably low mortality rates, similar to those associated with EVAR, in low-risk patients. This contrasts with EVAR, where the perioperative mortality rates remained relatively low as the risk score increased. Even high-risk patients with otherwise short life expectancy had an acceptably low perioperative mortality rate of 2.1% (95% CI, 0.8%-3.4%). In EVAR, anatomical factors such as small iliac vessels are associated with perioperative complications and may be more important than patients’ comorbidities.^22^ This finding is important as it suggests that EVAR may still be a valuable treatment option for high-risk patients, whereas OSR should likely be deferred. The risk categorization provided by the Society for Vascular Surgery guidelines from Eslami et al^23^ requires more clinical information for risk stratification and weights comorbidities equally in both OSR and EVAR.^1^ Furthermore, the validated risk estimation score by Eslami et al^23^ provides a probability of in-hospital mortality following elective AAA repair but no information on life expectancy.

Figure 2 demonstrates the challenges when analyzing long-term survival following OSR or EVAR. Although adjusted mortality rates were initially higher for OSR during the perioperative phase, they were higher for EVAR in the long term, resulting in crossing survival curves. Comparing RMST captures lifetime differences (area under the curve) and is effective with nonproportional hazards, as shown in Figure 2.^19,20^ In low-risk patients for whom perioperative mortality was similar for EVAR and OSR, survival curves diverged steadily during follow-up in favor of OSR (Figure 2A and Figure 3A). This led to an accumulated lifetime loss in the EVAR cohort and a higher mean survival time of 10 months for OSR restricted after 15 years (*P* = .02).

In all other risk groups, the excessive perioperative mortality for OSR led to an early survival advantage for EVAR (Figures 3B-3D). However, after 6 to 8 years, this benefit was gradually lost, particularly for low-to-moderate-risk and high-risk patients. The initial survival advantage of lower perioperative mortality with EVAR became nonsignificant after about 8.5 years and beyond. This study thus confirms the known trend for higher late mortality in the EVAR cohort among older patients (aged >70 years),^24^ those with severely impaired kidney function (eGFR <30 mL/min/1.73 m^2^), or those with COPD and an eGFR less than 60 mL/min/1.73 m^2^.^7,8,25^ The excess perioperative deaths among comorbid patients in the OSR cohorts (Figures 3B-3D) may contribute to later catch-up mortality in the EVAR cohorts, because many frail patients who survive EVAR may have a below-average life expectancy thereafter. However, this explanation does not account for low-risk patients, for whom perioperative mortality was similar but late mortality was higher following EVAR.

In moderate-to-high-risk patients (Figure 3C), the absence of excess late mortality after EVAR is surprising. Their survival pattern may resemble that of other risk groups, but this could be obscured by low patient numbers beyond 10 years of follow-up. Alternatively, residual confounding or a true difference may explain this finding.

This study emphasizes the potential in using risk stratification in patients with intact AAA. In low-risk patients, the association between treatment and the mean survival time was clinically relevant and statistically significant, favoring OSR. This is in line with the current European clinical practice guideline that recommends considering OSR as the preferred treatment modality in patients with long life expectancy (>10 to 15 years).^2^ Low-risk patients were defined as those younger than 70 years with an eGFR greater than or equal to 60 mL/min/1.73 m^2^, in case of coexistent COPD, or eGFR greater than or equal to 30 mL/min/1.73 m^2^ if no COPD was present.^7,8^

For all other patients with a life expectancy of less than 2 to 3 years, European guidelines recommend that EVAR should be considered as the preferred treatment modality if anatomically suitable.^2^ This study also support this recommendation: EVAR was associated with a lower perioperative mortality compared with OSR, and no difference in long-term survival was observed in low-to-moderate-risk and high-risk patients. In moderate-to-high-risk patients, the mean survival time even remained significantly higher for patients treated with EVAR throughout the entire follow-up of 12.5 years, supporting EVAR as the preferred treatment.

In patients with a limited life expectancy of less than 2 to 3 years, elective AAA repair is not recommended by current European guidelines.^2^ In clinical practice, balancing the potential benefits of elective AAA repair against the risks of rupture and estimated life expectancy is very challenging. High-risk patients in this study had a median life expectancy of approximately 5 years. In these patients, elective AAA repair should be cautiously approached, as it may offer no survival benefit and could even reduce life expectancy due to perioperative deaths. In this context, the current rigid diameter threshold may not be ideal, particularly given recent data suggesting that the risk of AAA rupture may be lower than previously reported.^26,27,28^ Adjusting the diameter threshold on the basis of patient’s risk profile could potentially reduce AAA-related mortality.^29^ The relatively simple risk score used in a previous study discriminated patients by their 10-year survival rate. In low-risk patients, the rates were 65.0% (95% CI, 61.4%-68.9%) for OSR and 51.9% (95% CI, 45.5%-59.1%) for EVAR.^7^ In contrast, 10-year survival rates were dramatically lower in high-risk patients, at 18.7% (95% CI, 13.8%-25.4%) for OSR and 7.8% (95% CI, 4.3%-14.2%) for EVAR, with survival rates for the intermediate-risk groups falling between these extremes.^7^ These findings highlight the risk score’s potential utility in estimating life expectancy during treatment planning. Conversely, the risk score used is not validated for estimating perioperative mortality where specific tools are already available. Besides the model of Eslami et al,^23^ the recently updated American College of Surgeons National Surgical Quality Improvement Program provides an online calculator for surgical risk.^30,31^ Depending on the planned procedure, this calculator estimates the risk of perioperative death as one of several outcome parameters. In addition to age and kidney and pulmonary function, the score requires 20 variables for precise estimates.

In the present study, the 10-year cumulative incidence of secondary ruptures was 2.6% (95% CI, 1.9%-3.4%) following OSR and 2.2% (95% CI, 1.7%-2.7%) following EVAR (*P* = .34). A Canadian study reported lower rates: 0.8% (95% CI, 0.6%-1.2%) for OSR and 1.5% (95% CI, 0.9%-2.5%) for EVAR (*P* = .69).^5^ Although the post-EVAR rupture rates align with expectations, the post-OSR rupture rate in our study is unexpectedly high. The *ICD-10* catalog lacks specific codes for ruptured iliac artery aneurysms, anastomotic pseudoaneurysms, or pararenal or renovisceral aneurysms, and our results may reflect true variations in late complications or variations in coding practices between the countries.

Radiation-induced cancer is a concern after infrarenal EVAR.^32^ At 10 years, solid tumor incidence was approximately 20%, with no significant difference between OSR and EVAR (18.6% [95% CI, 16.7%-20.5%] vs 20.5% [95% CI, 18.9%-22.1%]; *P* = .35). Combined with the 15.7% of patients who already had a tumor diagnosis at treatment, approximately one-third had cancer during the study. These rates align with the known risk of malignant neoplasm for 74-year-old individuals in northern Europe in 2020 (32.9% in male individuals, and 28.2% in female individuals).^33^ Of note, the cohort’s mean age was already 74.6 years at the time of treatment.

Further research is needed to understand the observed differences in stroke and heart failure rates. Potential explanations might be residual unmeasured confounding factors such as smoking or differences in the prescription habits and adherence to cardiovascular secondary prevention medications.

### Limitations

This cohort study has inherent limitations by its design and data source. First, diagnoses were derived from *ICD-10* codes rather than clinical records. The obtained survival curves are in line with several published studies, including those presented for the risk score used,^7,8^ but relevant clinical information, such as aneurysm diameter, smoking status, and the presence of clinical symptoms, was not available. Second, information on serum creatinine was missing for a considerable proportion of patients, predominantly in the earlier phase where OSR was more often offered, which carries the risk of a selection bias. Third, this study should not be seen as a recommendation for the choice of a particular surgical treatment method, but rather as supportive information when clinical decision-making is difficult because both surgical methods appear suitable.

## Conclusions

In this cohort study of 6891 patients with AAA, the potential benefits of risk stratification when planning treatment were highlighted. OSR was associated with higher perioperative mortality in all risk groups, but was associated with longer mean survival only in low-risk patients. Conversely, EVAR was associated with longer mean survival in moderate-to-high-risk patients. No differences in secondary AAA ruptures or malignant tumor diagnoses observed during follow-up between the 2 treatments.

## Notes

Supplement 1. **eTable 1.** Codes **eTable 2.** Baseline Characteristics of Raw and Weighted Low-Risk Cohort **eTable 3.** Baseline Characteristics of Raw and Weighted Low-to-Moderate Risk Cohort **eTable 4.** Baseline Characteristics of Raw and Weighted Moderate-to-High Risk Cohort **eTable 5.** Baseline Characteristics of Raw and Weighted High-Risk Cohort **eTable 6.** Raw and Weighted Survival Rates at 1, 5, and 10 years after Treatment **eFigure 1.** LASSO Variable Selection - Cross Validation **eFigure 2.** LASSO Variable Selection - Coefficient Paths **eFigure 3.** Inverse Probability Weighting **eFigure 4.** Cumulative Incidence of Diagnosis at 10 Years

Supplement 2.
Data Sharing Statement
