# Supplementary material for: Risk Stratification and Treatment Selection in Patients With Asymptomatic Abdominal Aortic Aneurysms
Source: JAMA Netw Open. 2025 Apr 7;8(4):e253559. doi: 10.1001/jamanetworkopen.2025.3559 (PMC11976495; doi:10.1001/jamanetworkopen.2025.3559)
Supplement: Supplement 1. — eTable 1. Codes eTable 2. Baseline Characteristics of Raw and Weighted Low-Risk Cohort eTable 3. Baseline Characteristics of Raw and Weighted Low-to-Moderate Risk Cohort eTable 4. Baseline Characteristics of Raw and Weighted Moderate-to-High Risk Cohort eTable 5. Baseline Characteristics of Raw and Weighted High-Risk Cohort eTable 6. Raw and Weighted Survival Rates at 1, 5, and 10 years after Treatment eFigure 1. LASSO Variable Selection - Cross Validation eFigure 2. LASSO Variable Selection - Coefficient Paths eFigure 3. Inverse Probability Weighting eFigure 4. Cumulative Incidence of Diagnosis at 10 Years [file jamanetwopen-e253559-s001.pdf]

Supplemental Online Content

Meuli L, Zimmermann A, Petersen JK, et al. Risk stratification and treatment selection in patients with asymptomatic abdominal aortic aneurysms. *JAMA Netw Open*. 2025;8(4):e253559. doi:10.1001/jamanetworkopen.2025.3559

eTable 1. Codes
eTable 2. Baseline Characteristics of Raw and Weighted Low-Risk Cohort
eTable 3. Baseline Characteristics of Raw and Weighted Low-to-Moderate Risk Cohort
eTable 4. Baseline Characteristics of Raw and Weighted Moderate-to-High Risk Cohort
eTable 5. Baseline Characteristics of Raw and Weighted High-Risk Cohort
eTable 6. Raw and Weighted Survival Rates at 1, 5, and 10 years after Treatment
eFigure 1. LASSO Variable Selection - Cross Validation
eFigure 2. LASSO Variable Selection - Coefficient Paths
eFigure 3. Inverse Probability Weighting
eFigure 4. Cumulative Incidence of Diagnosis at 10 Years

This supplemental material has been provided by the authors to give readers additional information about their work.

© 2025 Meuli L et al. *JAMA Network Open*.

eTable 1: Codes

Variable	Codes	
**Study population**		
Intact abdominal aortic aneurysm	ICD-10:	I71.4
Open surgical repair	SKS:	KPDG10, KPDG20, KPDG21, KPDG22, KPDG23, KPDG24, KPDG99, KPDN10
Endovascular repair	SKS:	KPDP10, KPDQ10, KPDQ20, KPDQ21
**Comorbidities**		
Chronic obstructive pulmonary disease (if ICD & ATC coding present)	ICD-10:<br>ICD-8:<br>ATC:	I27.8, I27.9, J40 - J47, J60 - J67, J68.4, J70.1, J70.3<br>490, 491, 492<br>R03
Dialysis	ICD-10:	Z99.2
Diabetes	ICD-10:<br>ICD-8:	E10 - 14<br>250
Atrial fibrillation	ICD-10:<br>ICD-8:	I48<br>42793, 42794
Coronary artery disease	ICD-10:<br>ICD-8:	I25<br>412
Myocardial infarction	ICD-10:	I21, I22
Heart failure	ICD-10:<br>ICD-8:	I42, I50, I11.0, I13.0, I13.2, J81.9<br>425, 428, 4270, 4271
Stroke	ICD-10:<br>ICD-8:	I63, I64<br>430 - 434, 436
Lymphoma	ICD-10:	C81 - C85, C88, C96, C90.0, C90.2
Solid tumor	ICD-10:	C00 - C26, C30 - C34, C37 - C41, C43, C45 - C58, C60 - C76, C97
Tumor metastasis	ICD-10:	C77 - C80
Ruptured abdominal aortic aneurysm	ICD-10:	I71.3

Caption eTable 1:

ICD-10: International classification of diseases, 10th revision.
https://icd.who.int/browse10/2019/en

ICD-8: International classification of diseases, 8th revision.
https://sundhedsdatastyrelsen.dk/-/media/sds/filer/rammer-og-retningslinjer/klassifikationer/sks-download/lukkede-klassifikationer/icd-8-klassifikation.txt?la=da

SKS: Sundhedsvæsenets Klassifikations System - Danish version of the Nordic Classification of Surgical Procedures (NCSP)
https://sundhedsdatastyrelsen.dk/da/rammer-og-retningslinjer/om-klassifikationer/sks-klassifikationer

ATC: Anatomical Therapeutic Chemical Classification
https://www.who.int/tools/atc-ddd-toolkit/atc-classification

© 2025 Meuli L et al. *JAMA Network Open*.

eTable 2: Baseline Characteristics of Raw and Weighted Low-Risk Cohort

	Patients, No. (%)			Patients, No. (%)		
	Raw Cohort			Weighted Cohort		
	Open Repair	EVAR		Open Repair	EVAR	
Variable	n = 1007	n = 431	Std. Diff.	n = 1417	n = 1482	Std. Diff.
**Age**, *years*	66.5 (2.6)[a]	66.8 (2.5)[a]	0.135	66.6 (2.6)[a]	66.4 (2.7)[a]	0.076
**Female sex**	167 (16.6)	49 (11.4)	0.151	217.7 (15.4)	221.7 (15.0)	0.011
**eGFR**, *ml/min/1.73m²*	72.0 (13.7)[a]	72.6 (14.5)[a]	0.046	72.4 (13.7)[a]	72.3 (14.2)[a]	0.009
**COPD**	63 (6.3)	51 (11.8)	0.195	104.2 (7.3)	135.9 (9.2)	0.066
**Atrial fibrillation**	59 (5.9)	37 (8.6)	0.105	94.5 (6.7)	103.3 (7.0)	0.012
**Coronary artery disease**	196 (19.5)	111 (25.8)	0.151	287.6 (20.3)	341.0 (23.0)	0.066
**Myocardial infarction**	134 (13.3)	72 (16.7)	0.095	207.5 (14.6)	214.9 (14.5)	0.004
**Heart failure**	40 (4.0)	53 (12.3)	0.308	64.7 (4.6)	144.3 (9.7)	0.202
**Diabetes**	76 (7.5)	54 (12.5)	0.166	117.6 (8.3)	163.3 (11.0)	0.092
**Stroke**	119 (11.8)	55 (12.8)	0.029	169.9 (12.0)	177.5 (12.0)	<0.001
**Solid Tumor**	97 (9.6)	77 (17.9)	0.241	166.2 (11.7)	182.9 (12.3)	0.019
**Lymphoma**	10 (1.0)	8 (1.9)	0.073	16.9 (1.2)	26.1 (1.8)	0.047
**Tumor metastasis**	≤5 (≤0.5)	8 (1.9)	0.139	5.7 (0.4)	20.5 (1.4)	0.104
**Date of treatment**[b]	03/2015 (60)	09/2016 (54)	0.340	10/2015 (59)	06/2015 (56)	0.063
2004 - 2008	131 (13.0)	14 (3.2)		154.3 (10.9)	90.8 (6.1)	
2009 - 2013	252 (25.0)	117 (27.1)		323.9 (22.8)	522.7 (35.3)	
2014 - 2018	366 (36.3)	145 (33.6)		520.9 (36.7)	470.5 (31.7)	
2019 - 2023	258 (25.6)	155 (36.0)		418.4 (29.5)	398.5 (26.9)	

Caption eTable 2:

Data were complete. Std. Diff. = Standardised mean difference between Open Repair and EVAR patients. The weighted cohort represents the adjusted population after inverse probability weighting (=pseudopopulation).

[a] Continuous variables are summarized with mean and standard deviation.

[b] Date of treatment was weighted as a continuous variable and presented with mean date and its standard deviation in brackets.

EVAR = Endovascular aneurysm repair; SMD = standardized mean difference; eGFR = estimated glomerular filtration rate; COPD = Chronic obstructive pulmonary disease.

© 2025 Meuli L et al. *JAMA Network Open*.

eTable 3: Baseline Characteristics of Raw and Weighted Low-to-Moderate Risk Cohort

	Patients, No. (%)			Patients, No. (%)		
	Raw Cohort			Weighted Cohort		
	**Open Repair**	**EVAR**		**Open Repair**	**EVAR**	
**Variable**	n = 1960	n = 1406	**Std. Diff.**	n = 3437	n = 3307	**Std. Diff.**
**Age**, *years*	74.5 (2.8)[a]	75.0 (2.9)[a]	0.175	74.8 (2.8)[a]	74.7 (2.9)[a]	0.030
**Female sex**	346 (17.7)	174 (12.4)	0.148	533.4 (15.5)	513.3 (15.5)	<0.001
**eGFR**, *ml/min/1.73m²*	72.4 (13.8)[a]	72.9 (13.9)[a]	0.032	72.6 (13.7)[a]	72.6 (14.0)[a]	0.002
**COPD**	17 (0.9)	15 (1.1)	0.020	28.9 (0.8)	34.2 (1.0)	0.020
**Atrial fibrillation**	180 (9.2)	188 (13.4)	0.133	363.4 (10.6)	377.4 (11.4)	0.027
**Coronary art. disease**	376 (19.2)	316 (22.5)	0.081	691.7 (20.1)	682.0 (20.6)	0.012
**Myocardial infarction**	221 (11.3)	211 (15.0)	0.111	425.3 (12.4)	439.4 (13.3)	0.027
**Heart failure**	100 (5.1)	105 (7.5)	0.098	225.5 (6.6)	192.2 (5.8)	0.031
**Diabetes**	161 (8.2)	147 (10.5)	0.077	304.9 (8.9)	294.5 (8.9)	0.001
**Stroke**	269 (13.7)	225 (16.0)	0.064	483.7 (14.1)	512.4 (15.5)	0.040
**Solid Tumor**	246 (12.6)	294 (20.9)	0.225	557.1 (16.2)	516.0 (15.6)	0.017
**Lymphoma**	23 (1.2)	16 (1.1)	0.003	50.9 (1.5)	33.1 (1.0)	0.043
**Tumor metastasis**	16 (0.8)	19 (1.4)	0.052	32.9 (1.0)	34.0 (1.0)	0.007
**Date of treatment**[b]	01/2016 (57)	07/2017 (51)	0.340	09/2016 (56)	07/2016 (53)	0.026
2004 - 2008	164 (8.4)	22 (1.6)		220.9 (6.4)	90.8 (2.7)	
2009 - 2013	471 (24.0)	290 (20.6)		705.8 (20.5)	894.6 (27.1)	
2014 - 2018	735 (37.5)	511 (36.3)		1282.9 (37.3)	1230.3 (37.2)	
2019 - 2023	590 (30.1)	583 (41.5)		1227.6 (35.7)	1091.3 (33.0)	

Caption eTable 3:

Data were complete. Std. Diff. = Standardised mean difference between Open Repair and EVAR patients. The weighted cohort represents the adjusted population after inverse probability weighting (=pseudopopulation).

[a] Continuous variables are summarized with mean and standard deviation.

[b] Date of treatment was weighted as a continuous variable and presented with mean date and its standard deviation in brackets.

EVAR = Endovascular aneurysm repair; SMD = standardized mean difference; eGFR = estimated glomerular filtration rate; COPD = Chronic obstructive pulmonary disease.

© 2025 Meuli L et al. *JAMA Network Open*.

eTable 4: Baseline Characteristics of Raw and Weighted Moderate-to-High Risk Cohort

	**Patients, No. (%)**			**Patients, No. (%)**		
	**Raw Cohort**			**Weighted Cohort**		
**Variable**	**Open Repair** n = 581	**EVAR** n = 635	**Std. Diff.**	**Open Repair** n = 1194	**EVAR** n = 1219	**Std. Diff.**
**Age**, *years*	77.9 (5.2)[a]	78.0 (5.0)[a]	0.412	78.8 (5.1)[a]	78.9 (5.7)[a]	0.021
**Female sex**	140 (24.1)	86 (13.5)	0.272	242.4 (20.3)	213.4 (17.5)	0.072
**eGFR**, *ml/min/1.73m²*	61.0 (20.3)[a]	68.4 (17.5)[a]	0.391	64.3 (18.9)[a]	65.1 (20.4)[a]	0.041
**COPD**	132 (22.7)	134 (21.1)	0.039	286.3 (24.0)	252.4 (20.7)	0.079
**Atrial fibrillation**	65 (11.2)	90 (14.2)	0.090	172.6 (14.5)	149.9 (12.3)	0.063
**Coronary art. disease**	136 (23.4)	145 (22.8)	0.014	302.0 (25.3)	271.4 (22.3)	0.071
**Myocardial infarction**	73 (12.6)	89 (14.0)	0.043	187.1 (15.7)	157.5 (12.9)	0.079
**Heart failure**	50 (8.6)	54 (8.5)	0.004	132.8 (11.1)	91.5 (7.5)	0.125
**Diabetes**	55 (9.5)	76 (12.0)	0.081	133.4 (11.2)	135.8 (11.1)	0.001
**Stroke**	75 (12.9)	105 (16.5)	0.102	150.0 (12.6)	195.3 (16.0)	0.099
**Solid Tumor**	66 (11.4)	142 (22.4)	0.297	192.9 (16.2)	217.8 (17.9)	0.046
**Lymphoma**	≤5 (≤0.9)	16 (2.5)	0.164	15.5 (1.3)	25.1 (2.1)	0.059
**Tumor metastasis**	≤5 (≤0.9)	10 (1.6)	0.065	7.7 (0.6)	17.9 (1.5)	0.081
**Date of treatment**[b]	12/2015 (60)	03/2018 (51)	0.481	11/2016 (57)	04/2017 (54)	0.088
2004 - 2008	59 (10.2)	18 (2.8)		84.5 (7.1)	57.5 (4.7)	
2009 - 2013	128 (22.0)	96 (15.1)		210.5 (17.6)	248.4 (20.4)	
2014 - 2018	212 (36.5)	205 (32.3)		439.4 (36.8)	407.2 (33.4)	
2019 - 2023	182 (31.3)	316 (49.8)		459.3 (38.5)	505.7 (41.5)	

Caption eTable 4:

Data were complete. Std. Diff. = Standardised mean difference between Open Repair and EVAR patients. The weighted cohort represents the adjusted population after inverse probability weighting (=pseudopopulation).

[a] Continuous variables are summarized with mean and standard deviation.

[b] Date of treatment was weighted as a continuous variable and presented with mean date and its standard deviation in brackets.

EVAR = Endovascular aneurysm repair; SMD = standardized mean difference; eGFR = estimated glomerular filtration rate; COPD = Chronic obstructive pulmonary disease.

© 2025 Meuli L et al. *JAMA Network Open*.

eTable 5: Baseline Characteristics of Raw and Weighted High-Risk Cohort

	Patients, No. (%)			Patients, No. (%)		
	Raw Cohort			Weighted Cohort		
	**Open Repair**	**EVAR**		**Open Repair**	**EVAR**	
**Variable**	n = 378	n = 493	**Std. Diff.**	n = 833	n = 901	**Std. Diff.**
**Age**, *years*	80.6 (4.6)[a]	81.7 (4.3)[a]	0.249	81.3 (4.6)[a]	81.3 (4.5)[a]	0.001
**Female sex**	86 (22.8)	96 (19.5)	0.080	152.3 (18.3)	206.8 (23.0)	0.116
**eGFR**, *ml/min/1.73m²*	43.4 (15.5)[a]	47.8 (14.0)[a]	0.295	44.6 (15.2)[a]	46.3 (14.7)[a]	0.117
**COPD**	104 (27.5)	152 (30.8)	0.073	246.0 (29.5)	252.4 (28.0)	0.033
**Atrial fibrillation**	54 (14.3)	88 (17.8)	0.097	131.9 (15.8)	133.5 (14.8)	0.028
**Coronary art. disease**	92 (24.3)	120 (24.3)	<0.001	224.9 (27.0)	206.7 (22.9)	0.093
**Myocardial infarction**	54 (14.3)	71 (14.4)	0.003	124.7 (15.0)	119.2 (13.2)	0.050
**Heart failure**	44 (11.6)	76 (15.4)	0.111	128.5 (15.4)	114.9 (12.8)	0.076
**Diabetes**	54 (14.3)	61 (12.4)	0.056	134.9 (16.2)	99.0 (11.0)	0.152
**Stroke**	70 (18.5)	80 (16.2)	0.061	154.2 (18.5)	136.9 (15.2)	0.088
**Solid Tumor**	52 (13.8)	107 (21.7)	0.209	159.4 (19.1)	156.5 (17.4)	0.046
**Lymphoma**	<5 (<1.3)	11 (2.2)	0.092	8.3 (1.0)	17.2 (1.9)	0.076
**Tumor metastasis**	<5 (<1.3)	<5 (<1.0)	0.090	<5 (<0.6)	<5 (<0.6)	0.081
**Date of treatment**[b]	04/2015 (65)	01/2018 (51)	0.572	08/2016 (62)	05/2017 (54)	0.154
2004 - 2008	55 (14.6)	10 (2.0)		84.6 (10.1)	32.0 (3.5)	
2009 - 2013	97 (25.7)	82 (16.6)		171.4 (20.6)	186.3 (20.7)	
2014 - 2018	109 (28.8)	173 (35.1)		241.3 (28.9)	322.5 (35.8)	
2019 - 2023	117 (31.0)	228 (46.2)		336.2 (40.3)	360.2 (40.0)	

Caption eTable 5:

Data were complete. Std. Diff. = Standardised mean difference between Open Repair and EVAR patients. The weighted cohort represents the adjusted population after inverse probability weighting (=pseudopopulation).

[a] Continuous variables are summarized with mean and standard deviation.

[b] Date of treatment was weighted as a continuous variable and presented with mean date and its standard deviation in brackets.

EVAR = Endovascular aneurysm repair; SMD = standardized mean difference; eGFR = estimated glomerular filtration rate; COPD = Chronic obstructive pulmonary disease.

© 2025 Meuli L et al. *JAMA Network Open*.

eTable 6: Raw and Weighted Survival Rates at 1, 5, and 10 years after Treatment

			Survival Rates at 1 Year (95%-CI)	
**Risk Group**	**No. of Deaths**	**No. at Risk**	**Raw Cohort**	**Weighted Cohort**
**Open Repair**				
Low	46	918	95.4 (94.1-96.7)	95.6 (94.3-96.8)
Low-to-moderate	182	1679	90.6 (89.3-91.9)	90.3 (88.9-91.7)
Moderate-to-high	92	461	84.0 (81.0-87.0)	83.6 (80.4-86.9)
High	95	269	74.7 (70.5-79.3)	76.7 (72.4-81.3)
Overall	415	3327	89.3 (88.4-90.3)	88.5 (87.4-89.6)
**EVAR**				
Low	20	384	95.2 (93.2-97.3)	96.1 (94.4-97.9)
Low-to-moderate	57	1226	95.8 (94.8-96.9)	96.0 (95.0-97.1)
Moderate-to-high	45	518	92.6 (90.5-94.7)	92.7 (90.5-95.0)
High	53	393	88.9 (86.1-91.8)	88.4 (85.4-91.5)
Overall	175	2521	93.9 (93.0-94.8)	94.5 (93.7-95.4)
			**Survival Rates at 5 Years (95%-CI)**	
**Risk Group**	**No. of Deaths**	**No. at Risk**	**Raw Cohort**	**Weighted Cohort**
**Open Repair**				
Low	145	624	83.7 (81.3-86.2)	83.6 (81.1-86.1)
Low-to-moderate	464	994	72.9 (70.8-75.0)	72.2 (70.0-74.5)
Moderate-to-high	212	232	58.5 (54.3-63.1)	56.5 (52.0-61.4)
High	180	113	46.5 (41.3-52.4)	48.4 (42.9-54.6)
Overall	1001	1963	71.1 (69.6-72.7)	69.1 (67.4-70.8)
**EVAR**				
Low	76	217	78.7 (74.5-83.1)	81.4 (77.4-85.7)
Low-to-moderate	298	599	72.0 (69.3-74.8)	73.4 (70.7-76.2)
Moderate-to-high	157	204	65.9 (61.5-70.6)	66.4 (61.9-71.3)
High	179	141	52.4 (47.3-58.0)	54.5 (49.4-60.3)
Overall	710	1161	68.6 (66.7-70.7)	71.8 (69.9-73.8)
			**Survival Rates at 10 Years (95%-CI)**	
**Risk Group**	**No. of Deaths**	**No. at Risk**	**Raw Cohort**	**Weighted Cohort**
**Open Repair**				
Low	252	239	65.0 (61.4-68.9)	64.7 (61.0-68.6)
Low-to-moderate	762	251	41.9 (38.9-45.1)	41.1 (38.1-44.4)
Moderate-to-high	308	53	27.6 (23.0-33.1)	26.1 (21.6-31.5)
High	231	24	18.7 (13.8-25.4)	18.8 (13.7-25.7)
Overall	1553	567	44.1 (42.1-68.9)	41.6 (39.6-43.8)
**EVAR**				
Low	129	75	51.9 (45.5-59.1)	54.9 (48.2-62.6)
Low-to-moderate	493	129	37.5 (33.7-41.8)	39.9 (35.9-44.4)
Moderate-to-high	251	21	19.4 (14.3-26.5)	23.3 (17.2-31.6)
High	260	8	7.8 (4.3-14.2)	8.8 (4.8-16.2)
Overall	1133	233	32.5 (29.9-35.4)	38.0 (35.0-41.2)

Captions eTable 6:
95%-CI = 95% confidence interval of survival rate; EVAR = Endovascular aortic repair.

© 2025 Meuli L et al. *JAMA Network Open*.

eFigure 1: LASSO Variable Selection - Cross Validation

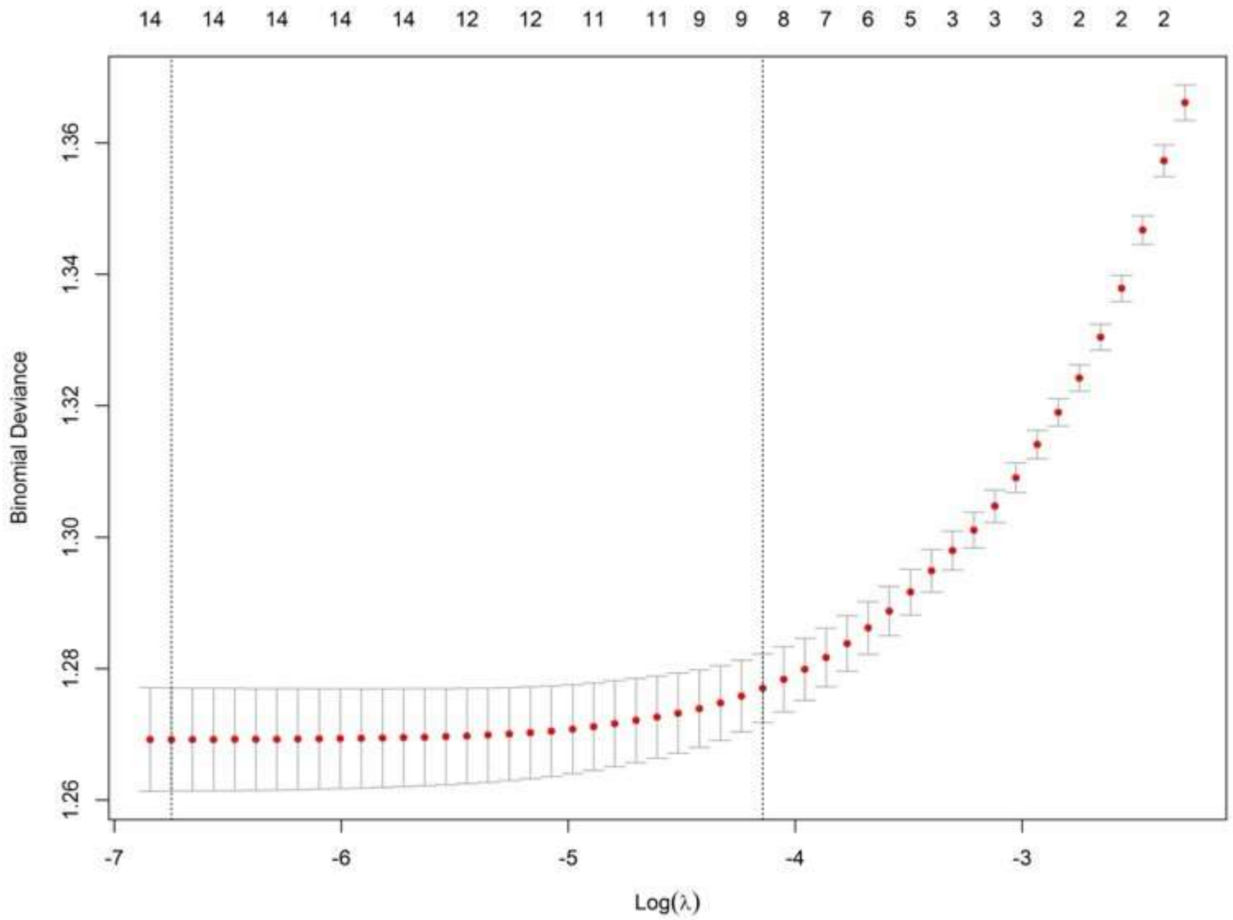

Caption eFigure 1:
Results of the LASSO variable selection process using the glmnet() R-package by Friedman et al., 2010.

Results of 10-fold cross-validation process with optimal number of variables in the model at λ_{1SE} (lambda) = 1 standard error of minimum λ. At λ_{1SE}, 9 of the 14 variables (numbers across the top) in the model had non-zero coefficients and were selected for the inverse probability weighting. The red dots indicate the estimated variance of the loss metric with 95% confidence intervals. The vertical lines show the locations of λ_{min} and λ_{1SE} of the minimum.

© 2025 Meuli L et al. *JAMA Network Open*.

eFigure 2: LASSO Variable Selection - Coefficient Paths

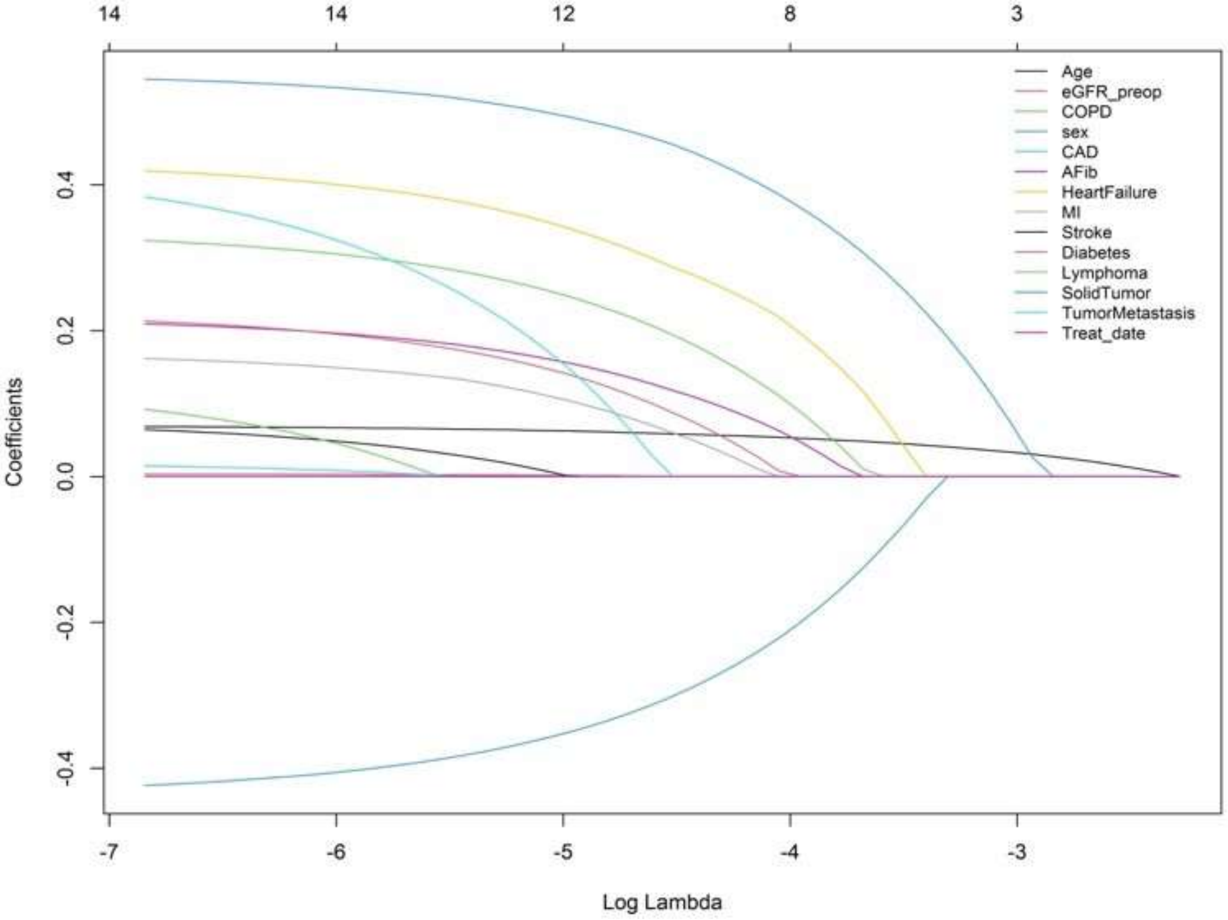

Caption eFigure 2:
Results of the LASSO variable selection process using the glmnet() R-package by Friedman et al., 2010.

Coefficient paths of the included variables against λ in the model during the shrinkage process. The axis above indicates the number of non-zero coefficients at the current log(λ), which is the effective degrees of freedom for the lasso.

© 2025 Meuli L et al. *JAMA Network Open*.

eFigure 3: Inverse Probability Weighting

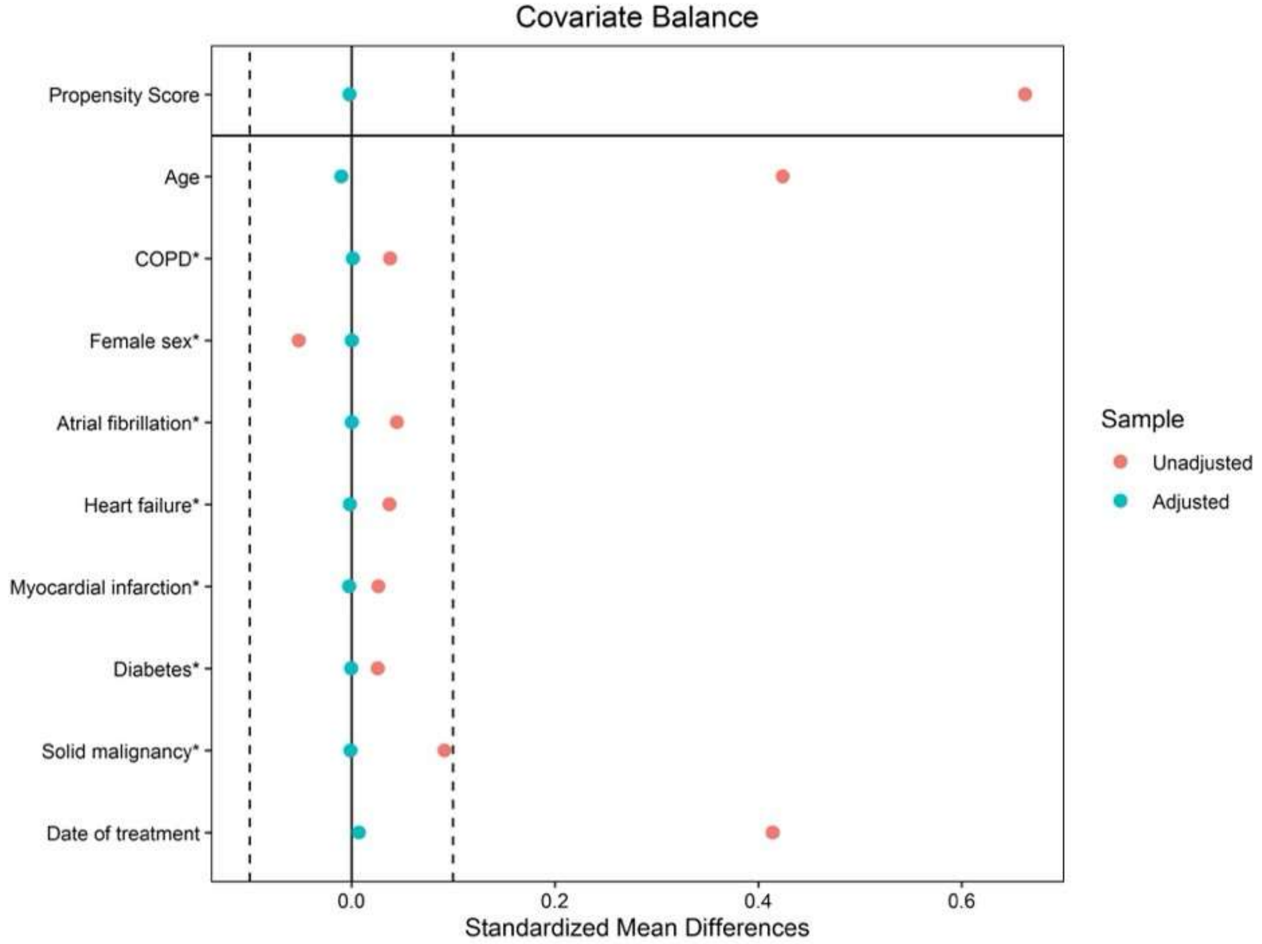

Caption eFigure 3:
Results of the IPW process using the cobalt() R-package by Noah Greifer, 2019.
Balance of covariates was assessed using standardized mean differences, with values <0.2 indicating good balance. The dashed lines are set at SME = +/- 0.1.
* Indicating binary variables.

© 2025 Meuli L et al. *JAMA Network Open*.

eFigure 4: Cumulative Incidence of Diagnosis at 10 Years

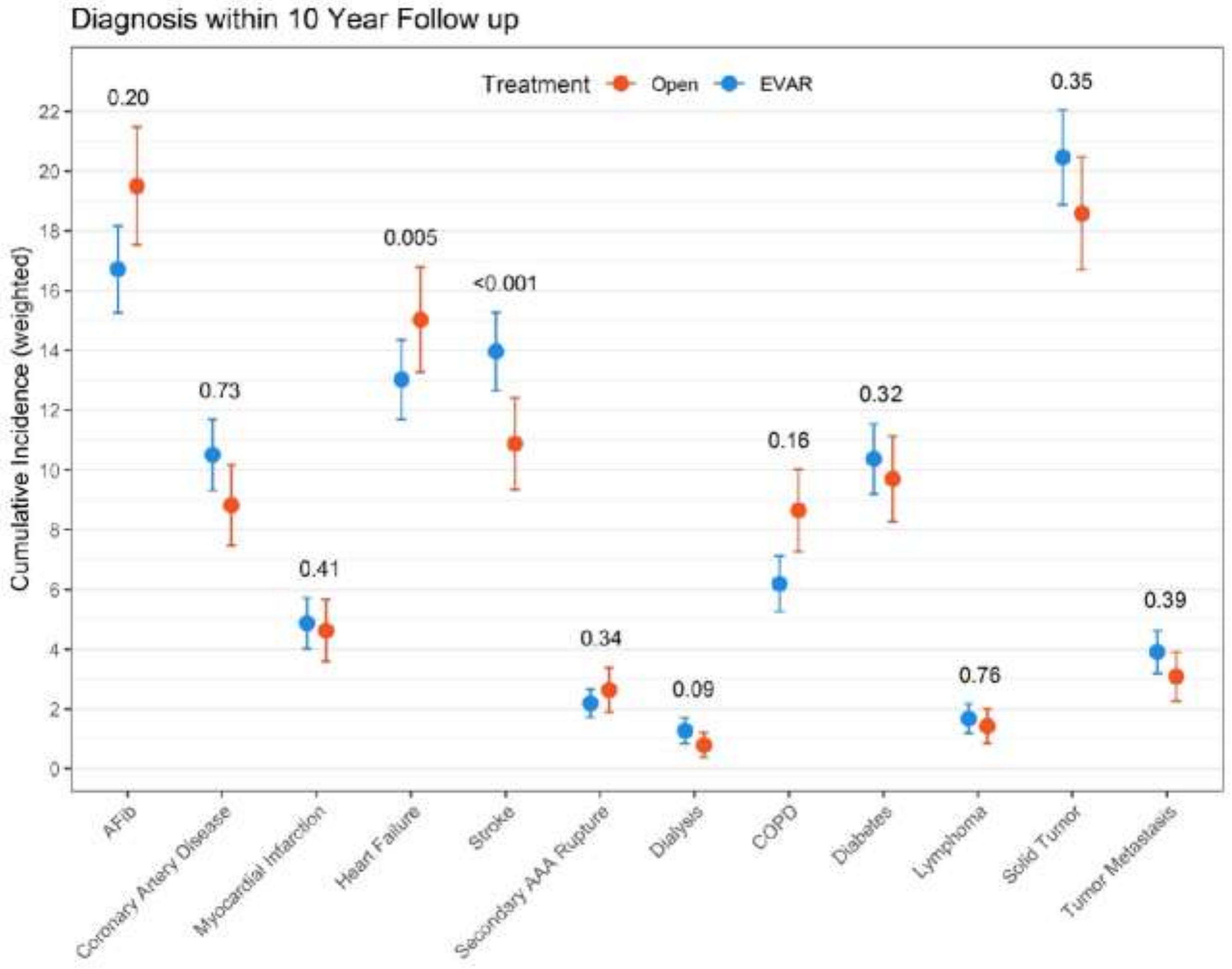

Footnote eFigure 4:

IPW-adjusted cumulative incidence of secondary diagnosis at 10 years after treatment in percent by treatment. Error bars indicate 95% confidence intervals. Death served as a competing risk. Rates were compared between treatments using Gray's tests. Raw data is available in the supplementary material. AFib = Atrial fibrillation; COPD = Chronic obstructive pulmonary disease.

© 2025 Meuli L et al. *JAMA Network Open*.
