# Supplementary material for: Risk Stratification and Treatment Selection in Patients With Asymptomatic Abdominal Aortic Aneurysms
Source: JAMA Netw Open. 2025 Apr 7;8(4):e253559. doi: 10.1001/jamanetworkopen.2025.3559 (PMC11976495; doi:10.1001/jamanetworkopen.2025.3559)
Supplement: Supplement 2. — Data Sharing Statement [file jamanetwopen-e253559-s002.pdf]

Data Sharing Statement

Meuli. Risk Stratification and Treatment Selection in Patients With Asymptomatic Abdominal Aortic Aneurysms. *JAMA Netw Open*. Published April 07, 2025.
doi:10.1001/jamanetworkopen.2025.3559

Data
Data available: No

Additional Information
Explanation for why data not available: All data were accessed in Statistics Denmark's research environment and are not publicly available. Researchers interested in accessing the data can directly apply to Statistics Denmark for further information or contact the corresponding author for guidance.
